# A dose-finding and safety study of novel erythropoiesis stimulating protein (NESP) for the treatment of anaemia in patients receiving multicycle chemotherapy

**DOI:** 10.1054/bjoc.2001.1748

**Published:** 2001-04

**Authors:** J Glaspy, J Singh Jadeja, G Justice, J Kessler, D Richards, L Schwartzberg, J Rigas, D Kuter, D Harmon, D Prow, G Demetri, D Gordon, J Arseneau, A Saven, H Hynes, R Boccia, J O'Byrne, A B Colowick

**Affiliations:** UCLA School of Medicine, 200 Medical Plaza, Suite 120, Los Angeles, CA 90024 USA; Hematology-Oncology Associates of Jacksonville, 5742 Booth Road, Jacksonville, FL 32207, US; Pacific Coast Hematology Oncology Medical Group, 11190 Warner Avenue, Suite 300, Fountain Valley, CA 92708, USA; Virginia Oncology Associates, 895 Middle Ground Blvd, Newport News, VA 2360, USA; Tyler Cancer Center, 910 East Houston, Tyler, TX 75702, USA; The West Clinic, 1775 Moriah Woods Boulevard, Suite 5, Memphis, TN 38117, USA; Dartmouth Medical School, Norris Cancer Center, Lebanon, NH 03756, USA; Massachusetts General Hospital, 100 Blossom Street, Boston, MA 02114, USA; Texas Cancer Care, Klabzuba Tower, 1300 W. Terrell, Suite 2060, Fort Worth, TX 76104, USA; Center for Sarcoma and Bone Oncology, Dana-Farber Cancer Institute and Harvard Medical School, 44 Binney Street, Boston, MA 02115, USA; San Antonio Tumor and Blood Clinic, 215 Quincy, Suite 314, San Antonio, TX 78215, USA; Albany Regional Cancer Center, 317 South Manning Boulevard, Suite 330, Albany, NY 12208, USA; Scripps Clinic, 10666 North Torrey Pines Road, La Jolla, CA 92037, USA; Cancer Center of Kansas, Suite 403, 818 North Emporia, Wichita, KS 67214, USA; Associates in Oncology and Hematology, 9707 Medical Center Drive, Shady Grove Medical Building, Rockville, MD, USA; Amgen Inc, One Amgen Center Drive, Thousand Oaks, CA 91329, USA

**Keywords:** anaemia, cancer, chemotherapy, darbepoetin alfa, solid tumours, transfusion

## Abstract

Darbepoetin alfa is a novel erythropoiesis stimulating protein (NESP), which stimulates erythropoiesis by the same mechanism as recombinant human erythropoietin (rHuEPO). NESP has been shown to be safe and efficacious in patients with chronic renal failure. NESP is biochemically distinct from rHuEPO, due to its increased sialic acid content. NESP has an approximately 3-fold greater half-life. rHuEPO has been shown to be safe and effective for the treatment of chemotherapy-induced anaemia. This study assessed the safety and efficacy of NESP administered once per week, under the supervision of a physician, to patients with solid tumours who were receiving multicycle chemotherapy for up to 12 weeks. Three dose cohorts are presented in this sequential, unblinded and dose-escalating study. Thirteen to 59 patients received NESP (0.5, 1.5 or 2.25 mcg kg^−1^wk^−1^) in each cohort. Patients were monitored for adverse events, including antibody formation to NESP and for effects on haemoglobin. NESP appeared to be well tolerated. Adverse events were similar across all cohorts and were consistent with the population being studied. No antibody formation was detected over the 16-week study period and follow-up. A dose–response relationship was evident for NESP and multiple measures of efficacy, including proportion of patients responding to NESP and the mean change in haemoglobin by week 4 and end of treatment for NESP 0.5, 1.5 and 2.25 mcg kg^−1^wk^−1^cohorts (mean change in haemoglobin at end of treatment was 1.24, 1.73 and 2.15 g dl^−1^respectively). Controlled studies of this agent at higher doses and less frequent schedules of administration are ongoing. © 2001 Cance Cancer Research Campaign


*British Journal of Cancer* (2001) **84** (Supplement 1), 17–23
© 2001 Cancer Research Campaign
doi: 10.1054/ bjoc.2001.1748, available online at http://www.idealibrary.com on IDEAL®

### A dose-finding and safety study of novel erythropoiesis stimulating protein (NESP) for the treatment of anaemia in patients receiving multicycle chemotherapy

**J Glaspy[1], J Singh Jadeja[2], G Justice[3], J Kessler[4], D Richards[5], L Schwartzberg[6], J Rigas[7], D Kuter[8], D Harmon[8], D Prow [9], G Demetri[10], D Gordon[11], J Arseneau[12], A Saven[13], H Hynes[14], R Boccia[15], J O'Byrne[16] and AB Colowick[16]**

[1]UCLA School of Medicine, 200 Medical Plaza, Suite 120, Los Angeles, CA 90024, USA; [2]Hematology-Oncology Associates of Jacksonville, 5742 Booth Road, Jacksonville, FL 32207, USA; [3]Pacific Coast Hematology Oncology Medical Group, 11190 Warner Avenue, Suite 300, Fountain Valley, CA 92708, USA; [4]Virginia Oncology Associates, 895 Middle Ground Blvd, Newport News, VA 2360, USA; [5]Tyler Cancer Center, 910 East Houston, Tyler, TX 75702, USA; [6]The West Clinic, 1775 Moriah Woods Boulevard, Suite 5, Memphis, TN 38117, USA; [7]Dartmouth Medical School, Norris Cancer Center, Lebanon, NH 03756, USA; [8]Massachusetts General Hospital, 100 Blossom Street, Boston, MA 02114, USA; [9]Texas Cancer Care, Klabzuba Tower, 1300 W. Terrell, Suite 2060, Fort Worth, TX 76104, USA; [10]Center for Sarcoma and Bone Oncology, Dana-Farber Cancer Institute and Harvard Medical School, 44 Binney Street, Boston, MA 02115, USA; [11]San Antonio Tumor and Blood Clinic, 215 Quincy, Suite 314, San Antonio, TX 78215, USA; [12]Albany Regional Cancer Center, 317 South Manning Boulevard, Suite 330, Albany, NY 12208, USA; [13]Scripps Clinic, 10666 North Torrey Pines Road, La Jolla, CA 92037, USA; [14]Cancer Center of Kansas, 818 North Emporia, Suite 403, Wichita, KS 67214, USA; [15]Associates in Oncology and Hematology, Shady Grove Medical Building, 9707 Medical Center Drive, Rockville, MD, USA; [16]Amgen Inc, One Amgen Center Drive, Thousand Oaks, CA 91329, USA

**Summary** Darbepoetin alfa is a novel erythropoiesis stimulating protein (NESP), which stimulates erythropoiesis by the same mechanism as recombinant human erythropoietin (rHuEPO). NESP has been shown to be safe and efficacious in patients with chronic renal failure. NESP is biochemically distinct from rHuEPO, due to its increased sialic acid content. NESP has an approximately 3-fold greater half-life. rHuEPO has been shown to be safe and effective for the treatment of chemotherapy-induced anaemia. This study assessed the safety and efficacy of NESP administered once per week, under the supervision of a physician, to patients with solid tumours who were receiving multicycle chemotherapy for up to 12 weeks. Three dose cohorts are presented in this sequential, unblinded and dose-escalating study. Thirteen to 59 patients received NESP (0.5, 1.5 or 2.25 mcg $kg^{-1}$ $wk^{-1}$) in each cohort. Patients were monitored for adverse events, including antibody formation to NESP and for effects on haemoglobin. NESP appeared to be well tolerated. Adverse events were similar across all cohorts and were consistent with the population being studied. No antibody formation was detected over the 16-week study period and follow-up. A dose–response relationship was evident for NESP and multiple measures of efficacy, including proportion of patients responding to NESP and the mean change in haemoglobin by week 4 and end of treatment for NESP 0.5, 1.5 and 2.25 mcg $kg^{-1}$ $wk^{-1}$ cohorts (mean change in haemoglobin at end of treatment was 1.24, 1.73 and 2.15 g $dl^{-1}$ respectively). Controlled studies of this agent at higher doses and less frequent schedules of administration are ongoing. © 2001 Cancer Research Campaign

**Keywords**: anaemia; cancer; chemotherapy; darbepoetin alfa; solid tumours; transfusion

Patients with cancer receiving multicycle chemotherapy are frequently anaemic. The aetiology of anaemia in these patients is multifactorial. In addition to the myelosuppressive effects of chemotherapy, direct effects on the renal tubules by these agents, particularly platinum-based compounds, leads to a decrease in the production of erythropoietin (EPO). This hormone is responsible for terminal differentiation, proliferation and survival of red blood cell precursors (Koury and Bondurant, 1990). Patients with cancer have also been shown to have inappropriately low levels of circulating EPO for their degree of anaemia, reflecting a perturbation in this homeostatic mechanism (Miller et al, 1990). This latter problem may be due to the inflammatory state associated with malignancy, a form of the anaemia of chronic disease (Means, 1995; Jelkmann, 1998). Other causes of decreased erythropoiesis in patients with cancer include radiation therapy, nutritional deficiencies, and replacement of the bone marrow with tumour cells. In addition to the direct mechanical effect of tumour cells in the bone marrow, their elaboration or induction of inflammatory cytokines, such as tumour necrosis factor (TNF) and interleukin-1 (IL-1), can lead to a myelophthisic process associated with fibrosis or fatty replacement of the marrow (Argiles and Lopez-Soriano, 1999). Finally, bleeding from the tumour bed or bleeding due to a systemic coagulopathy can contribute to anaemia in patients with cancer. Anaemia is associated with a plethora of symptoms, including dyspnoea and fatigue. Fatigue is the most-often reported symptom in patients with cancer and has been found to have severe detrimental effects on their lives (Curt, 2000). Although fatigue often results from multiple factors in these patients, anaemia represents a common and treatable aetiology. Studies show a relationship among low haemoglobin values, fatigue and poor quality of life in patients with cancer (Cella, 1998).

If a patient develops severe or symptomatic anaemia, red blood cell transfusions may be required, with their attendant risks to patients with cancer. Acute transfusion reactions can occur, and although the blood supply is safer with respect to infection than previously, risk of transmission of infectious agents still exists (Walker, 1987; Goodnough et al, 2000). In addition, there is some concern that frequent red blood cell transfusions with allogeneic

*Correspondence to:* J Glaspy

This study was supported by Amgen Inc, Thousand Oaks, California.

blood may adversely effect the immune system of patients with cancer, thereby increasing the tendency to develop infections, hastening the time to relapse or shortening survival (Cascinu et al, 1994; Goodnough et al, 2000).

Recombinant human erythropoietin (rHuEPO) is effective in preventing and ameliorating treatment-related anaemia in patients with cancer (Abels, 1992; Maraveya and Pettengell, 1998). In a placebo-controlled study in patients with cisplatin-associated anaemia, blood transfusions were required less often in patients receiving rHuEPO 150 U $kg^{-1}$ three times a week than in patients receiving placebo (20% vs 56%) (Abels, 1992). Similar results were observed in patients receiving nonplatinum-based chemotherapy (Abels, 1992). Large community-based studies examining the effectiveness of rHuEPO in cancer patients have generally shown response rates in the range of 50 to 60% (Glaspy et al, 1997; Demetri et al, 1998). One of these studies (Glaspy et al, 1997) of 2030 patients demonstrated a mean (standard deviation (SD)) increase in haemoglobin over baseline levels of 1.8 (2.1) g $dl^{-1}$. Despite the extensive clinical experience with rHuEPO in the oncology setting, the dose response, rapidity of the response and pharmacokinetics of rHuEPO are not fully understood in this population of patients.

Darbepoetin alfa is a novel erythropoiesis stimulating protein (NESP) that stimulates erythropoiesis in the same manner as rHuEPO. NESP is biochemically distinct from rHuEPO, created by site-directed mutagenesis that results in 2 more glycosylation sites allowing for additional sialic acid. These changes significantly increase in vivo biologic activity and are associated with a 3-fold greater terminal half-life (Egrie et al, 1997). Extensive studies in patients with chronic renal failure have confirmed this 3-fold increase in half-life, allowing it to maintain haemoglobin levels as well as rHuEPO despite being administered less frequently (Macdougall et al, 1999). The prolonged half-life has been observed in cancer patients undergoing multiple cycles of chemotherapy (Glaspy et al, 2000), predicting that it need be administered once per week or less. Preliminary data suggest the possibility of administering NESP once every 3 weeks so that its dosing can be synchronized with once every 3 week chemotherapy regimens (Kotasek et al, 2000).

This paper presents partial results from an ongoing dose-escalation study of NESP in patients undergoing chemotherapy. The primary objective of this study is to assess the safety of NESP administered by subcutaneous injection to anaemic patients with solid tumours who were receiving multicycle chemotherapy. In addition, this study was designed to determine clinically effective doses of NESP administered weekly in this setting.

### PATIENTS AND METHODS

### Patients

The institutional review boards of the participating medical centres approved the protocol and all patients gave written informed consent before any study-related procedures were performed.

Patients were eligible for study enrolment if they were at least 18 years of age; had a solid tumour and had at least 12 additional weeks of cyclic chemotherapy planned; were anaemic (haemoglobin ≤ 11.0 g $dl^{-1}$), primarily because of their cancer or chemotherapy; and if they had adequate serum folate and vitamin $B_{12}$ levels and renal and hepatic functions. Patients were to be excluded if they had overt bleeding or haemolysis, primary or metastatic malignancy of the central nervous system; had received more than two red blood cell transfusions within 28 days before start of study or had received any red blood cell transfusion within 2 weeks of start of study; had received rHuEPO therapy within 8 weeks of start of study or any previous treatment with NESP; were pregnant, breast-feeding, or not using adequate birth control measures; or had a history of seizure disorder, active cardiac disease, hypertension or a primary haematologic disorder as the cause of their present anaemia.

### Study design

This is an ongoing phase 1–2, multicentre, open-label, sequential dose-escalation study in anaemic patients with solid tumours who are receiving multicycle chemotherapy. The partial results reported here represent 3 dose cohorts who were treated weekly and for whom the final results are available.

Eligible patients received one of 3 doses of NESP under the supervision of a physician (0.5, 1.5 or 2.25 mcg $kg^{-1}$ $wk^{-1}$) as a subcutaneous injection starting on the first day of chemotherapy. Enrolment to the next higher cohort was initiated when a minimum of 4 patients had safely completed at least 6 weeks of treatment, as determined by a safety monitoring committee. After safety had been determined for the 2.25 mcg $kg^{-1}$ $wk^{-1}$ cohort, patients were enrolled into the 1.5 mcg $kg^{-1}$ $wk^{-1}$ cohort to better define the dose–response curve. Study drug was administered under the supervision of a physician for up to 12 weeks.

### Study drug

Darbepoetin alfa (ARANESP™, Amgen Inc, Thousand Oaks, CA) is a novel erythropoiesis stimulating protein (NESP), formulated with human serum albumin. Since this was a dose-finding study, patients receiving NESP were not allowed to increase the dose if their haemoglobin concentrations remained stable or decreased.

### Treatment procedure

Patients were monitored for vital signs and had complete blood counts every week while on study. Eastern Cooperative Oncology Group (ECOG) performance status, blood chemistries, serum iron, iron-binding capacity, ferritin and transferrin saturation were measured at various time points during the study. Testing for antibodies to NESP and coagulation panels were done at specified time points during the study.

At any time during the study, the dose of NESP was withheld if a patient's haemoglobin level increased to > 15.0 g $dl^{-1}$ (for men) or > 14.0 g $dl^{-1}$ (for women). NESP was reinstated at the next lowest dose level, or reduced by 50% if the patient was receiving the lowest dose level, once the haemoglobin decreased to ≤ 13.0 g $dl^{-1}$. NESP was administered at this adjusted dose for the rest of the study. Originally, the dose of NESP was reduced to the next lowest dose level if the patient had a rapid rate of rise of haemoglobin (i.e. an increase ≥ 2.0 g $dl^{-1}$ over any 28-day period in the absence of a red blood cell transfusion). The criterion for dose reduction was removed during the conduct of the study due to lack of safety concern. Cohorts affected by this change are described in the results section.

© 2001 Cancer Research Campaign

### Safety measurements

The primary endpoint of the study was the safety of NESP, which was assessed in terms of adverse events and antibody formation. Serum was collected before study drug administration to provide a baseline for the antibody-screening assay and then at regular intervals throughout the study. The screening assay was a radio-immunoprecipitation assay used routinely in NESP studies in the oncology and nephrology settings. Rate of rise of haemoglobin and maximum haemoglobin values were calculated. Changes in concomitant medications, laboratory tests and vital signs were also examined. All patients who received at least one dose of study drug were evaluable for the safety analysis.

### Efficacy measurements

The potential efficacy of NESP was assessed based on the number and proportion of patients in each treatment group who achieved a haemoglobin response, as measured by an increase in haemoglobin of ≥ 2.0 g dl$^{-1}$ from baseline in the absence of red blood cell transfusions in the previous 28 days during the 12-week treatment phase. The maximum change in haemoglobin from baseline during the first 4 weeks of NESP treatment and at the end of 12 weeks of study was assessed. In addition, the incidence of red blood cell transfusions during week 5 through week 12 was calculated.

### Statistical analysis

The proportion of patients achieving a haemoglobin response was calculated using the Kaplan–Meier method. All patients who withdrew from study early and who had not met the criteria for response at the time of study withdrawal were censored on the day of their last haemoglobin measurement as a non-responder.

The proportion of patients with cancer who achieve a haemoglobin response when treated with rHuEPO is historically 50 to 60% (Abels, 1992). Therefore, a clinically effective dose was defined as a dose at which ≥ 50% of patients achieved a haemoglobin response, ≤ 20% of patients in the safety analysis set had clinical sequelae associated with haemoglobin that exceeds the highest acceptable level (> 14.0 g dl$^{-1}$ for women, > 15.0 g dl$^{-1}$ for men), and ≤ 20% of patients experienced a dose-limiting toxicity. The maximum change in haemoglobin from baseline during the first 4 weeks of NESP therapy and the 12-week study period was determined as another measurement of efficacy.

Data from studies of rHuEPO indicate the effects on red blood cell transfusion requirements are not apparent until the second month of treatment (Abels, 1992). Therefore, the proportion of patients receiving red blood cell transfusions during weeks 5 to 12 was determined.

### RESULTS

### Subject disposition

One hundred and seven patients have received NESP in 3 dose cohorts (0.5, 1.5 and 2.25 mcg kg$^{-1}$ wk$^{-1}$) for whom final data are available. Forty (38%) of the patients receiving NESP terminated the study prematurely. The most common reasons for early termination included withdrawal of consent and death. Sixty-seven (63%) of the patients completed the 12-week treatment and 4-week follow-up portion of the study.

**Table 1** Patient baseline demographics and clinical characteristics

| | NESP (mcg kg$^{-1}$ wk$^{-1}$) | | | |
|---|---|---|---|---|
| | 0.5 (*n* = 13) | 1.5 (*n* = 35) | 2.25 (*n* = 59) | All (*n* = 107) |
| Age, years | | | | |
| Mean (SD) | 61.3 (13.3) | 62.5 (12.2) | 61.8 (10.3) | 62.0 (11.2) |
| Sex, *n* (%) | | | | |
| Female | 9 (69.2%) | 25 (71.4%) | 42 (71.2%) | 76 (71.0%) |
| Male | 4 (30.8%) | 10 (28.6%) | 17 (28.8%) | 31 (29.0%) |
| Primary disease site, *n* (%) | | | | |
| Breast | 2 (15.4%) | 11 (31.4%) | 18 (30.5%) | 31 (29.0%) |
| Lung | 1 (7.7%) | 2 (5.7%) | 11 (18.6%) | 14 (13.1%) |
| Gastrointestinal | 3 (23.1%) | 9 (25.7%) | 14 (23.7%) | 26 (24.3%) |
| Gynaecologic | 5 (38.5%) | 4 (11.4%) | 4 (6.8%) | 13 (12.1%) |
| Genitourinary | | 4 (11.4%) | 1 (1.7%) | 5 (4.7%) |
| Other solid tumour | 2 (15.4%) | 5 (14.3%) | 11 (18.6%) | 18 (16.8%) |
| ECOG performance, *n* (%) | | | | |
| 0 | 3 (23.1%) | 12 (34.3%) | 21 (35.6%) | 36 (33.6%) |
| 1 | 7 (53.8%) | 22 (62.9%) | 33 (55.9%) | 62 (57.9%) |
| 2 | 2 (15.4%) | 1 (2.9%) | 5 (8.5%) | 8 (7.5%) |
| Hgb, g dl$^{-1}$ | | | | |
| Mean (SD) | 9.82 (1.03) | 9.72 (1.07) | 9.97 (1.01) | 9.87 (1.03) |
| Median | 9.70 | 9.70 | 10.10 | 10.00 |
| Min, Max | 8.3, 11.4 | 7.0, 11.5 | 6.9, 12.0 | 6.9, 12.0 |
| Endogenous EPO, mU ml$^{-1}$ | | | | |
| Mean (SD) | 50.14 (36.28) | 37.52 (22.84) | 46.13 (45.32) | 43.80 (38.48) |
| Median | 44.30 | 31.76 | 29.80 | 31.76 |
| Min, Max | 12.0, 123.2 | 12.0, 101.8 | 12.0, 251.1 | 12.0, 251.1 |

ECOG = Eastern Cooperative Oncology Group; EPO = erythropoietin; Hgb = haemoglobin; SD = standard deviation.

© 2001 Cancer Research Campaign 

### Demographic and baseline characteristics

Baseline demographic and subject characteristics are given in Table 1. The mean age of patients was 62.0 years; 71% of the patients were women. Breast cancer was the most common malignancy, followed by gastrointestinal and lung malignancies. More than 80% of the patients in all groups had a performance score ≤ 1 based on the ECOG scale.

Patients were required to have a haemoglobin level of ≤ 11.0 g $dl^{-1}$ at screening to participate in this study. The mean (SD) haemoglobin was 9.87 (1.03) g $dl^{-1}$. Since patients received NESP up to 11 days after screening, some had a baseline haemoglobin > 11.0 g $dl^{-1}$.

### Study drug administration

A total of 107 patients in 3 dose cohorts received at least one dose of NESP. The protocol stipulated that NESP be reduced if a patient exceeded a haemoglobin threshold (14.0 g $dl^{-1}$ for women and 15.0 g $dl^{-1}$ for men) or if the haemoglobin increased more than 2.0 g $dl^{-1}$ over any 28-day period. When it was determined that a rapid rise in haemoglobin was not associated with any apparent safety concerns, the latter criterion was removed after the first 30 patients in the 2.25 mcg $kg^{-1}$ $wk^{-1}$ cohort had completed treatment with NESP. The remaining patients in the 2.25 mcg $kg^{-1}$ $wk^{-1}$ cohort and all the patients in the 1.5 mcg $kg^{-1}$ $wk^{-1}$ cohort were enrolled after this dose reduction criterion had been removed. The actual average dose delivered in each of the 3 cohorts is given in Table 2. Only the 2.25 mcg $kg^{-1}$ $wk^{-1}$ cohort had a meaningful reduction in the average dose delivered, with a mean of 2.07 mcg $kg^{-1}$ $wk^{-1}$ administered.

### Safety results

The primary endpoint of the study was safety, specifically measured by report of adverse events and antibody formation for NESP. Six patients (6%) died on study and six (6%) discontinued study because of adverse events. The five most commonly reported adverse events were fatigue (44%), nausea (32%), diarrhoea (29%), vomiting (25%), and anorexia (21%), all common complaints of patients receiving cytotoxic chemotherapy. Sixteen (15%) patients reported adverse events that were determined to be treatment-related by the investigators. These events included injection-site pain (7%), fever (2%), pain (2%) and limb pain (2%). Twenty-eight (26%) patients reported a serious adverse event; one (rectal bleeding) was attributed by the investigator to study drug. Clinically meaningful trends were not apparent in laboratory values. No relationship between the rate of rise of haemoglobin, reports of hypertension as an adverse event or anti-hypertensive medication was apparent. Similarly, no relationship was observed between the rate of rise of haemoglobin and the incidence or severity of any specific adverse event. An analysis was done correlating the maximum change in haemoglobin to the maximum change in blood pressure within the 14-day window of the change in haemoglobin. As shown in Figure 1, there is no appreciable correlation between these two parameters, suggesting that in this population, changes in haemoglobin do not adversely affect blood pressure.

No antibodies to NESP were detected and no clinical sequela indicative of antibody formation was observed.

### Efficacy results

The percentage of patients who met the definition of haemoglobin response (≥ 2.0 g $dl^{-1}$ increase over baseline) increased as the dose of study drug increased, with 3 (23%) in the 0.5 mcg $kg^{-1}$ $wk^{-1}$ group, 12 (44%) in the 1.5 mcg $kg^{-1}$ $wk^{-1}$ group and 29 (52%) in the 2.25 mcg $kg^{-1}$ $wk^{-1}$ group (Table 2).

The mean change in haemoglobin from baseline was calculated for the first 4 weeks and at the end of the 12-week treatment period (Table 2). The mean values increased as the dose of NESP

**Table 2** Patient haemoglobin response to NESP and actual dose delivered

| | NESP (mcg $kg^{-1}$ $wk^{-1}$) | | |
|---|---|---|---|
| | **0.5** | **1.5** | **2.25** |
| Subjects treated, *n* | 13 | 35 | 59 |
| Haemoglobin response | | | |
| Number of patients | 3 | 12 | 29 |
| Kaplan–Meier percents (95% CI) | 23 (0, 46) | 44 (25, 63) | 52 (39, 66 |
| Maximum change from baseline haemoglobin, g $dl^{-1}$ | | | |
| Through week 4, mean (95% CI) | 0.26 (0.04, 0.48) | 0.63 (0.40, 0.86) | 0.82 (0.60, 1.04) |
| Through entire treatment, mean (95% CI) | 1.24 (0.72, 1.75) | 1.73 (1.21, 2.25) | 2.15 (1.77, 2.52) |
| RBC transfusions from week 5 to end of treatment | | | |
| Number of patients | 2 | 7 | 7 |
| Percent of patients (95% CI) | 15 (2, 45) | 20 (8, 37) | 12 (5, 23) |
| Dose of study drug received | | | |
| Number | 13 | 35 | 59 |
| Mean | 0.48 | 1.51 | 2.07 |
| SD | 0.05 | 0.01 | 0.31 |
| Median | 0.50 | 1.51 | 2.24 |
| Q1, Q3 | 0.50, 0.51 | 1.50, 1.52 | 1.88, 2.25 |
| Min, Max | 0.36, 0.51 | 1.47, 1.54 | 1.27, 2.29 |

CI = confidence interval; SD = standard deviation.

*© 2001 Cancer Research Campaign*

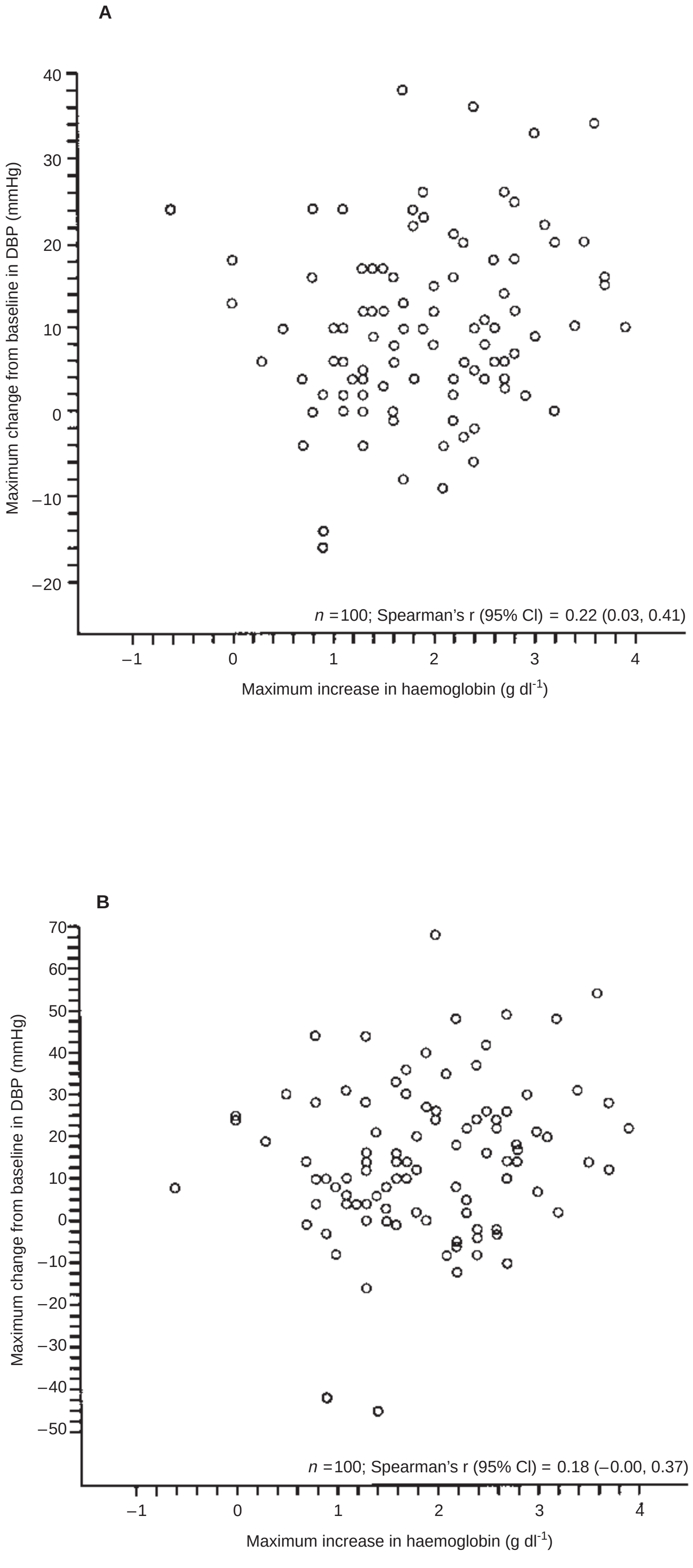

**Figure 1** The maximum increase between any two haemoglobin values within any 28-day period from first dose onwards plotted against the maximum change from baseline blood pressure that occurred within 14 days of the maximum increase in haemoglobin. Increases in haemoglobin that occurred within 28 days of a red blood cell transfusion were not considered when calculating the maximum. Panel **A** is a plot of diastolic blood pressure (DBP) and panel **B** is a plot of systolic blood pressure (SBP)

© 2001 Cancer Research Campaign increased at both the 4-week and the 12-week endpoints, suggesting a potential dose-dependent relationship for this efficacy parameter.

Overall, a relatively low rate of blood transfusion was observed in this study (Table 2). Two (15%) patients receiving 0.5 mcg $kg^{-1}$ $wk^{-1}$, 7 (20%) receiving 1.5 mcg $kg^{-1}$ $wk^{-1}$, and 7 (12%) receiving 2.25 mcg $kg^{-1}$ $wk^{-1}$ required a red blood cell transfusion during weeks 5 to 12 of the study.

### DISCUSSION

Anaemia is an increasingly recognized cause of morbidity in patients with cancer who are undergoing chemotherapy. The use of rHuEPO in this setting has increased dramatically over the last few years, as the association between improvement in haemoglobin levels and improvement in quality of life and a reduction in fatigue has been recognized (Glaspy et al, 1997; Demetri et al, 1998; Gabrilove et al, 1999; Cleeland et al, 1999; Littlewood et al, 2000). Although much has been learnt regarding the potential to benefit patients through the use of erythropoietic agents, several questions remain including the optimal dose and schedule to maximize patient benefit; the role of oral and parenteral iron supplementation in maximizing response; and the impact of successful therapy on outcomes of chemotherapy and radiotherapy.

This study is the first published report regarding the use of NESP, the first of a new generation of erythropoiesis stimulating proteins with increased half-life, in the chemotherapy-induced anaemia setting. These are the partial results of an ongoing trial aimed at characterizing dose response and rapidity of response and assessing the safety profile of NESP in this setting.

The results from this study suggest that NESP is safe when administered to patients with anaemia who are undergoing chemotherapy. The adverse event profile was dominated by predictable findings (e.g. fatigue, nausea) in this population of patients with primarily advanced malignancy receiving multicycle chemotherapy. No unexpected trends were noted in the incidence or severity of adverse events. The issue of the rate of rise in haemoglobin and its association with adverse events was investigated. No relationship was apparent. Specifically, hypertension and thrombotic events, two adverse events of historical interest, were not differentially reported for patients with a more rapid increase in haemoglobin. These observations are consistent with the physiologic differences between cancer patients and those with chronic renal failure such as volume sensitivity, reduced homeostasis of blood pressure control and a high incidence of hypertension seen in patients with renal failure. NESP appears to be safe at all doses tested to date. No antibodies to NESP were detected in this study.

The efficacy of NESP was assessed in 3 ways. First, the proportion of patients who achieved a 2.0 g $dl^{-1}$ increase in haemoglobin over baseline was investigated. A dose-dependent increase in the proportion of patients meeting this criterion was apparent. Historically, rHuEPO has generally been associated with a 50 to 60% response rate (Abels, 1992; Glaspy et al, 1997; Demetri et al, 1998). These studies allowed for the dose of rHuEPO to be doubled during the 6 to 8 weeks of the study if patients were not exhibiting a response. No such dose adjustment was allowed for the NESP doses in this study. Since the protocol stipulated dose reductions when patients reached various haemoglobin thresholds, the average delivered dose of NESP was less than that assigned to the 2.25 mcg $kg^{-1}$ $wk^{-1}$ cohort. These patients received, on average, only 2.07 mcg $kg^{-1}$ $wk^{-1}$ of NESP. Whether higher response rates are possible with increasing doses of NESP is the subject of ongoing studies.

Another efficacy parameter that was assessed in this study was the mean (SD) change in haemoglobin during the first 4 weeks and the entire 12 weeks of the study. Again, a dose-dependent relationship was suggested, with the greatest changes being associated with the highest dose. The magnitude of the changes in this study at higher dose levels seems consistent with that observed in the literature (Abels, 1992; Glaspy et al, 1997) and suggests a drug effect.

Finally, the incidence of red blood cell transfusions was assessed throughout the study. Since the literature suggests that rHuEPO does not influence the incidence of transfusions during the first month of therapy (Abels, 1992), the period of week 5 to week 12 was analysed. No relationship to dose across the 3 cohorts was apparent with respect to this outcome; however, in general, the transfusion rates were low.

Since the data presented are partial results from an ongoing study, limited conclusions can be drawn. First, the lack of control data makes it difficult to generalize these findings to other patient populations, or to the published data with respect to rHuEPO. The outcome of the higher doses studied seems to be comparable to what would be expected with rHuEPO, but patient selection could have confounding influences on this determination. A dose-dependent relationship with efficacy parameters (e.g. proportion of patients responding and mean change in haemoglobin) is apparent, suggesting that these findings are due to the effects of the study drug. Although the design of this study allows for evaluation of the 3 specified doses, it does not provide information that would allow extrapolation of the effect of increasing the dose of NESP in patients who do not exhibit a haemoglobin response, as is the standard for rHuEPO. The dose-dependent increase in haemoglobin outcomes suggests that such a dose increase may lead to higher response rates, but this speculation must be confirmed in a study designed to assess this endpoint. Finally, although rHuEPO is currently approved for administration three times per week in the United States, it is standardly being administered at 40 000 U $wk^{-1}$ (Gabrilove et al, 1999). The 3-fold increase in the half-life of NESP compared with rHuEPO as demonstrated in the nephrology setting was confirmed in the oncology setting (Heatherington et al, 2001) and suggests that NESP can be administered less frequently. Schedules should be explored in which NESP is given less frequently than the weekly schedule reported in this study, and higher doses of NESP need to be explored to further investigate the dose–response relationship in a well-controlled study.

### ACKNOWLEDGEMENTS

Elizabeth Faust, PhD; Susan Armstrong; Suzana Giffin, PharmD; and MaryAnn Foote, PhD, assisted in writing this paper.

### REFERENCES

Abels RI (1992) Use of recombinant human erythropoietin in the treatment of anemia in patients who have cancer. *Semin Oncol* **19**: 29–35

Argiles JM and Lopez-Soriano FJ (1999) The role of cytokines in cancer cachexia. *Med Res Rev* **19**: 223–248

Cascinu S, Fedeli A, Del Ferro E, Luzi Fedeli S and Catalano G (1994) Recombinant human erythropoietin treatment in cisplatin-associated anemia: a randomized double-blind trial with placebo. *J Clin Oncol* **12**: 1058–1062

Cella D (1998) Factors influencing quality of life in cancer patients: anemia and fatigue. *Semin Oncol* **25**: 43–46

 © 2001 Cancer Research Campaign

Cleeland CS, Demetri GD and Glaspy J (1999) Identifying hemoglobin levels for optimal quality of life: results of an incremental analysis. *Proc Am Soc Clin Oncol* **18**: 574a (abstract 2215)
Curt GA (2000) Impact of fatigue on quality of life in oncology patients. *Semin Hematol* **37**: 14–17
Demetri GD, Kris M, Wade J, Degos L and Cella D (1998) Quality-of-life benefit in chemotherapy patients treated with epoetin alfa is independent of disease response or tumor type: results from a prospective community oncology study. Procrit Study Group. *J Clin Oncol* **16**: 3412–3425
Egrie JC, Dwyer E, Lykos M, Hitz A and Browne JK (1997) Novel erythropoiesis stimulating protein (NESP) has a longer serum half-life and greater in vivo biological activity than recombinant human erythropoietin (rHuEPO). *Blood* **90**: 56a–57a (abstract 243)
Gabrilove JL, Einhom LH, Livingston RB, Winer E and Cleeland CS (1999) Once-weekly dosing of Epoetin alfa is similar to three times weekly dosing in increasing hemoglobin and quality of life. *Proc Am Soc Clin Oncol* **18**: 574a (abstract 2216)
Glaspy J, Bukowski R, Steinberg D, Taylor C, Tchekmedyian S and Vadhan-Raj S (1997) Impact of therapy with epoetin alfa on clinical outcomes in patients with nonmyleoid malignancies during cancer chemotherapy in community oncology practice. Procrit Study Group. *J Clin Oncol* **15**: 1218–1234
Glaspy J, Colowick AB and Heatherington A (2000) Novel erythropoiesis stimulating protein (NESP) exhibits a prolonged serum half-life ($T_{1/2}$) in oncology patients (pts). *Proc Am Soc Clin Oncol* **19**: 54a (abstract 210)
Goodnough LT, Skikne B and Brugnara C (2000) Erythropoietin, iron, and erythropoiesis. *Blood* **96**: 823–833
Heatherington AC, Schuller J and Mercer AJ (2001) Pharmacokinetics of novel erythropoiesis stimulating protein (NESP) in cancer patients: preliminary report. *Br J Cancer* **84** (Supp 1): 11–16
Jelkmann W (1998) Proinflammatory cytokines lowering erythropoeitin production. *J Interferon Cytokine Res* **18**: 555–559
Kotasek D, The ARANESP 980291 Study Group, Berg R, Poulsen E and Colowick A (2000) Randomized, double-blind, placebo controlled phase I/II dose finding study of ARANESP™ administered once every three weeks in solid tumor patients. *Blood* **96**: 294a–295a (abstract 1268)
Koury MJ and Bondurant MC (1990) Control of red cell production: the roles of programmed cell death (apoptosis) and erythropoeitin. *Transfusion* **30**: 673–674
Littlewood TJ, Rapoport B and Bajetta E (2000) Possible relationship of hemoglobin levels with survival in anemic cancer patients receiving chemotherapy. *Proc Am Soc Clin Oncol* **19**: 605a (abstract 2381)
Macdougall IC, Gray S, Elston O, Breen C, Jenkins B, Browne J and Egrie J (1999) Pharmacokinetics of novel erythropoiesis stimulating protein compared with Epoetin alfa in dialysis patients. *J Am Soc Nephrol* **10**: 2392–2395
Maraveya S and Pettengell R (1998) What is the role of erythropoietin in patients with solid tumors? *Ann Oncol* **9**: 239–241
Means RT Jr (1995) Pathogenesis of the anemia of chronic disease: a cytokine-mediated anemia. *Stem Cells* **13**: 32–37
Miller CB, Jones RJ, Piantadosi S, Abeloff MD and Spivak JL (1990) Decreased erythropoietin response in patients with anemia of cancer. *N Engl J Med* **332**: 1689–1692
Walker R (1987) Transfusion risks. *Am J Clin Pathol* **88**: 371–378

*© 2001 Cancer Research Campaign* 
